# Blood–Brain Barrier Penetration of Novel 4-Trifluoromethyl-Coumarin Hybrids with Antibacterial Properties as Potential Brain Therapeutics in the Context of Spatially Diverse Healthcare Systems

**DOI:** 10.3390/ijms26199655

**Published:** 2025-10-03

**Authors:** Paweł Kowalczyk, Dominik Koszelewski, Tomasz Misztal, Michał Szlis, Patrycja Młotkowska, Marcin Gołębiewski, Krzysztof Głowacz, Malwina Kocot, Michał Marczyk, Aleksandra Wypych, Apoloniusz Kurylczyk, Anna Krajewska-Pędzik, Ryszard Ostaszewski

**Affiliations:** 1 The Kielanowski Institute of Animal Physiology and Nutrition, Polish Academy of Sciences, Instytucka 3, 05-110 Jabłonna, Poland; t.misztal@ifzz.pl (T.M.); m.szlis@ifzz.pl (M.S.); p.mlotkowska@ifzz.pl (P.M.); 2 Institute of Organic Chemistry, Polish Academy of Sciences, Kasprzaka 44/52, 01-224 Warsaw, Poland; dominik.koszelewski@icho.edu.pl; 3 Institute of Animal Sciences, Warsaw University of Life Sciences, Nowoursynowska 161, 02-787 Warsaw, Poland; marcin_golebiewski@sggw.edu.pl (M.G.); krzysztof_glowacz@sggw.edu.pl (K.G.); 4 Faculty of Management and Organization, Silesian University of Technology, 41-800 Zabrze, Poland; malwakocot@gmail.com; 5 Department of Data Science and Engineering, Silesian University of Technology, 44-100 Gliwice, Poland; michal.marczyk@polsl.pl; 6 Yale Cancer Center, Yale School of Medicine, New Haven, CT 06510, USA; 7 Centre for Modern Interdisciplinary Technologies, Nicolaus Copernicus University in Toruń, Wileńska 4, 87-100 Toruń, Poland; wypych@umk.pl; 8 Institute of Spatial Management and Socio-Economic Geography, University of Szczecin, 70-453 Szczecin, Poland; apoloniusz.kurylczyk@usz.edu.pl; 9 Institute of Physical Culture Sciences, University of Szczecin, Piastow 40b/6, 71-065 Szczecin, Poland; anna.krajewska@usz.edu.pl

**Keywords:** coumarins, peptidomimetics, imines, antimicrobial activity, cerebrospinal fluid, *Staphylococcus aureus*, *Escherichia coli*

## Abstract

Effective treatment of central nervous system (CNS) infections remains a major challenge, as most therapeutic agents do not efficiently cross the blood–brain barrier (BBB) and the blood–cerebrospinal fluid barrier (BCSFB). Coumarin derivatives are of particular interest due to their broad pharmacological activity, favorable safety profile, and potential to penetrate biological barriers. Eight novel coumarin-based peptidomimetics functionalized with trifluoromethyl or methyl scaffolds were synthesized and evaluated as antimicrobial agents with the ability to cross the blood–brain barrier. Antimicrobial activity of the investigated compounds was tested against *Staphylococcus aureus* and multiple *Escherichia coli* strains (K12, R2, R3, R4) using minimum inhibitory concentration (MIC) and minimum bactericidal concentration (MBC) assays. Cytotoxicity was assessed in vitro in BALB/c-3T3 mouse fibroblasts and αT3-1 pituitary gonadotrope cells using the MTT assay. In vivo studies were performed in sheep to assess transfer of the compounds from blood to cerebrospinal fluid (CSF). All synthesized derivatives demonstrated antimicrobial activity and acceptable cytotoxicity, comparable to those of clinically used antibiotics. CF_3_-modified coumarin peptidomimetics show promise as antimicrobial agents with the potential to penetrate the BBB/BCSFB. These findings support further investigation of coumarin-based scaffolds as a platform for the development of novel therapeutics for CNS infections.

## 1. Introduction

Encephalitis can develop as a consequence of viral, bacterial, fungal, or protozoan infections, or as a result of immune system dysfunction [1,2,3]. Bacterial meningitis represents one of the most severe forms of neuroinfection, characterized by inflammation of the meningeal structures, such as the arachnoid and subarachnoid space. It is typically caused by pathogens such as *Streptococcus agalactiae* (group B *streptococci*), *Bacillus* spp., *Neisseria meningitidis*, or *Streptococcus pneumonia* [4,5,6]. Current treatment strategies are primarily based on intravenous administration of antibiotics, supplemented with antipyretics and analgesics. Despite advances in therapy, bacterial infections of the central nervous system (CNS) remain a major public health challenge [7,8,9]. Even with rapid and adequate treatment, meningitis can cause irreversible neurological damage, long-term CNS dysfunction, or death. One of the main obstacles in the treatment of CNS infections is the limited penetration of many antibacterial agents across the blood–brain barrier (BBB) [7,8,9]. The BBB is a semipermeable barrier formed by endothelial cells of the brain microvasculature, sealed by extensive tight junctions [8,9,10,11]. This unique structure protects the CNS from bloodborne toxins and pathogens, but also severely restricts the passage of therapeutic compounds. Generally, only small lipophilic molecules with molecular weights below 400 to 600 Da can passively diffuse across the BBB [12,13,14,15,16]. Therefore, drug lipophilicity, molecular size, and substituent chemistry are key factors in the design of CNS-active agents [17,18,19]. However, most conventional chemical drugs and biopharmaceuticals are effectively excluded from entering the brain through the BBB and the blood–cerebrospinal fluid barrier (BCSFB) [20,21]. In addition to its barrier role, the choroid plexus regulates the molecular exchange between the blood and brain and produces cerebrospinal fluid (CSF), further influencing drug bioavailability in the CNS. Although the integrity of the BBB is essential for neuroprotection, it limits antibiotic access to the brain [21,22,23,24,25,26]. Disruption of barrier function, while pathological, can temporarily increase permeability and facilitate the penetration of antibiotics, but in many cases direct administration to the CSF remains necessary. Impaired BBB function is also involved in hypoxic–ischemic brain injury, neuroinflammation, and ischemia–reperfusion injury, which are accompanied by increased transport into the CNS [23,27,28,29,30]. Given these limitations, alternative therapeutic strategies have been explored, including direct systemic administration, combination therapies, and the design of novel compounds with optimized physicochemical properties. Coumarin-based compounds are of particular interest. Coumarins exhibit strong pharmacological activity, low toxicity, broad therapeutic spectra, and favorable bioavailability, while also showing reduced susceptibility to resistance. They have been investigated for anti-inflammatory, antioxidant, analgesic, antithrombotic, and antimicrobial effects. Importantly, coumarin derivatives have demonstrated antibacterial activity against key neurotropic pathogens and may exhibit the ability to cross the BBB, making them promising candidates for human and veterinary medicine [31,32,33,34,35,36,37,38,39]. Recent studies emphasize the importance of functionalizing coumarins with substituents that enhance the permeability of the BBB [40,41]. Among these, the trifluoromethyl group has been highlighted as a modification capable of improving lipophilicity and facilitating CNS penetration.

The social and economic consequences of BBB/BCSFB dysfunction compel us to seek and develop new methods of prevention and early diagnosis to detect disorders and risk factors for neurodegenerative diseases such as Alzheimer’s, Parkinson’s, and encephalopathy. Although the role of psychological factors in the development and progression of central nervous system diseases has been studied for many years in rats [23,40], there is a lack of research on the brains of mammals, such as sheep. Research into the causes of neurodegenerative diseases leads to the development of new, more effective treatments and therapies that can improve patients’ quality of life.

Therefore, in the present study, we synthesized and evaluated several coumarin-based peptidomimetic derivatives functionalized with a trifluoromethyl- (CF_3_-) functional group.

## 2. Results and Discussion

Our working hypothesis was that coumarin-based peptidomimetic derivatives would exhibit antibacterial activity and, at least partially, penetrate the BBB after intravenous administration. Coumarin-based compounds have been consistently reported to exhibit a considerable propensity for blood–brain barrier (BBB) penetration. Multiple studies indicate that structural modifications, particularly hydroxyl substitutions or the introduction of multifunctional ligands, enhance the ability of coumarin derivatives to cross the BBB while retaining favorable drug-likeness profiles compatible with oral administration. In silico ADMET (absorption, distribution, metabolism, excretion, toxicity) analyses further support the notion that numerous coumarin derivatives display physicochemical properties conducive to BBB permeability without violating established drug-likeness criteria [42]. Notably, multifunctional coumarin derivatives developed as anti-Alzheimer’s agents have demonstrated effective BBB penetration in combination with antioxidant and metal-chelating activities [43,44]. Structural features, such as hydroxyl substitution on the coumarin nucleus, are particularly associated with enhanced antioxidant potential and may contribute to improved BBB transport [45]. Evidence for BBB penetration is frequently derived from molecular docking and pharmacokinetic modeling studies, which confirm the brain-targeting potential of these compounds [46]. Furthermore, conjugation of coumarin scaffolds with established pharmacophores, such as donepezil, has yielded derivatives capable of crossing the BBB and exerting selective effects on central nervous system (CNS) enzymes [47]. Therefore, coumarin derivatives represent a structurally versatile and pharmacologically promising class of molecules with demonstrated potential for CNS drug development. Their ability to penetrate the BBB, particularly when optimized through rational design strategies, underscores their utility as scaffolds for the development of therapeutics targeting neurological disorders [48].

**Figure 1:**
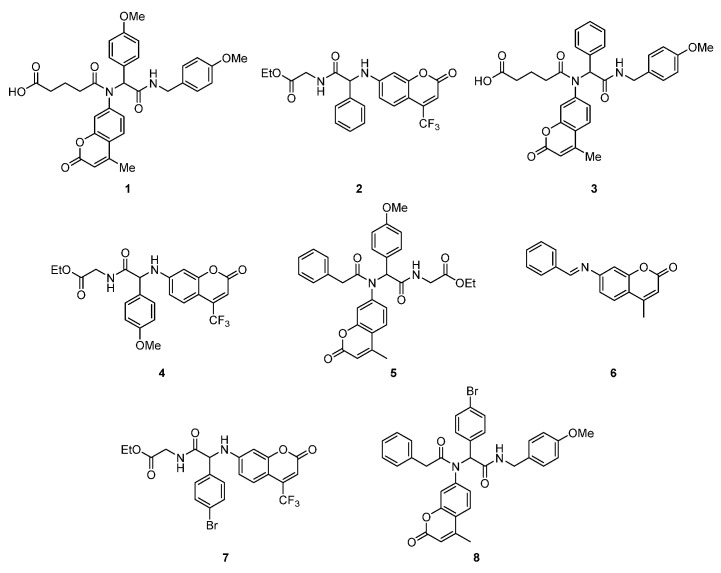
Investigated coumarin derivatives **1**–**8**.

To achieve our goal, we have designed and prepared series of structurally diversified peptidomimetics (Figure 1). Further, to evaluate our hypothesis, we conducted a series of biological studies. First, in vitro assays were performed to assess bactericidal activity against selected endogenous and pathogenic bacterial strains, including Staphylococcus aureus (ATCC 23235), Escherichia coli K12 (ATCC 25404), and its R2 (ATCC 39544), R3 (ATCC 11775), and R4 (ATCC 39543) mutants [34,35,36]. Minimum inhibitory concentrations (MIC) and minimum bactericidal concentrations (MBCs) were determined. Second, in vivo studies in sheep were carried out. The sheep represented a large animal model with CNS anatomy and physiology comparable to humans that could be used to assess the transfer of coumarin derivatives from blood to CSF using intraventricular cannulation for continuous CSF sampling [49,50,51,52,53,54]. Finally, in vitro toxicity was evaluated in mammalian cell lines, including BALB/c-3T3 fibroblasts and αT3-1 pituitary gonadotropes, using the MTT assay [55].

### 2.1. In Vitro Experiment—Bactericidal Effects

#### 2.1.1. Cytotoxic Studies of the Library of Coumarin with Trifluoromethyl Group

The bactericidal activity of compounds **1**–**8** (Figure 1) was evaluated in bacterial cells following a previously established protocol [56].

**Figure 2:**
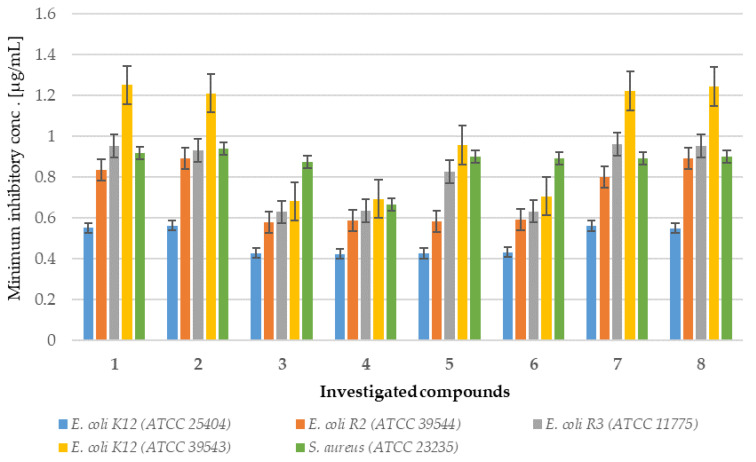
Minimum inhibitory concentration (MIC) of the coumarin derivatives **1**–**8** investigated in selected bacterial strains.

**Figure 3:**
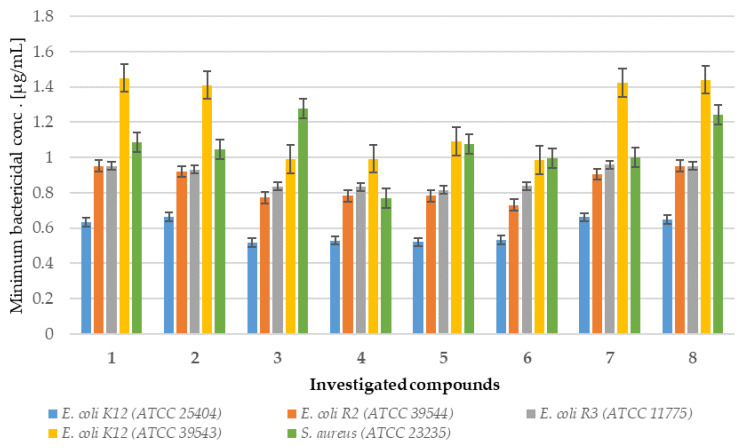
Minimum bactericidal concentration (MBC) of the coumarin derivatives **1**–**8** investigated in selected bacterial strains.

**Figure 4:**
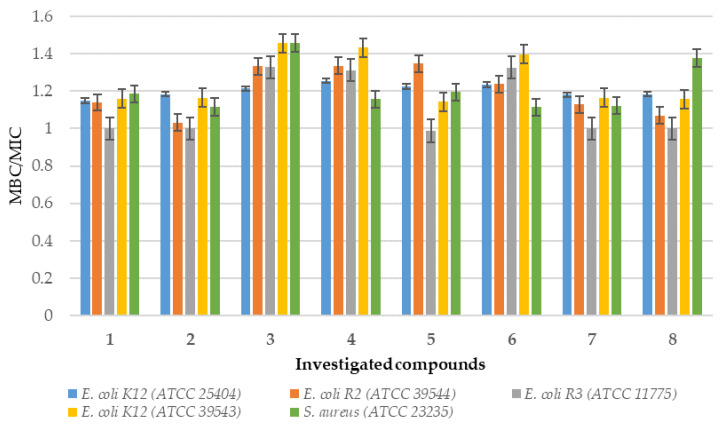
The MBC/MIC ratio of the coumarin derivatives **1**–**8** investigated in selected bacterial strains.

**Figure 5:**
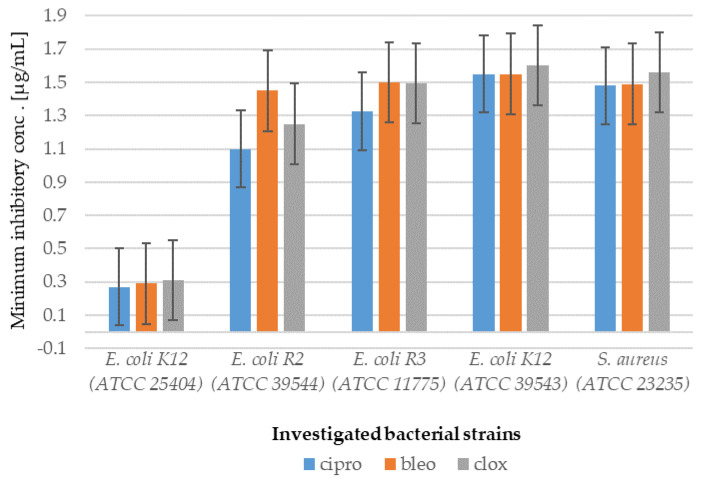
Examples of MIC with a bacterial model strain in selected strains of *E. coli* and *S. aureus* for studying antibiotics ciprofloxacin (cipro), bleomycin (bleo) and cloxacillin (clox).

Analysis of MIC and MBC assays revealed that the investigated coumarin derivatives exhibited MIC values ranging from 0.58 to 1.25 µg/mL against model strains *Escherichia coli* (K12 (ATCC 25404), R2 (ATCC 39544), R3 (ATCC 11775), and R4 (ATCC 39543)) and *Staphylococcus aureus* (ATCC 23235). The corresponding MBC values were within the range of 0.62–1.43 µg/mL for both species (Figure 2, Figure 3, Figure 4 and Figure 5). The minimal inhibitory concentration (MIC) reflects the lowest concentration of a compound that inhibits visible bacterial growth, while the minimal bactericidal concentration (MBC) represents the lowest concentration required to achieve bactericidal activity rather than merely bacteriostatic effects.

MIC and MBC assays were performed using model strains of *E. coli* and *S. aureus* cultured in 48-well plates and treated with synthesized coumarin derivatives, including trifluoromethyl substituted analogues. All of the compounds tested induced visible colorimetric changes, with intensity varying according to dilution. Among the strains tested, *E. coli* demonstrated higher sensitivity than *S. aureus*, which can be attributed to the presence of lipopolysaccharide (LPS) as the major structural component of its outer membrane. In particular, significant effects were observed at dilutions of 10^−3^, corresponding to 0.58 µg/mL. LPS, which consists of multiple acyl chains forming a hydrophobic base and numerous sugar residues within the hydrophilic core and O-antigen domains [18], plays a key role in the natural defense mechanisms of bacteria under adverse environmental conditions. Antimicrobial agents are generally classified as bactericidal or bacteriostatic agents. When the MBC/MIC ratio is 4–6, the compound is considered bactericidal, indicating the ability to achieve concentrations that eliminate 99.9% of the bacterial population. In contrast, a higher MBC/MIC ratio suggests a bacteriostatic effect, where safe therapeutic concentrations may inhibit growth but are insufficient to achieve bactericidal activity (Figure 2 and Figure 3). For many compounds, however, this classification remains context-dependent, influenced by achievable drug concentrations in the target tissue and the specific characteristics of the pathogen.

#### 2.1.2. Analysis of *E. coli* R2–R4 Strains Treated with Investigated Compounds **1**–**8**

The results of the present study (MIC values), together with previously reported findings [31,56], demonstrate that CF_3_-substituted coumarin exhibits pronounced toxic effects against the *E. coli* and *S. aureus model strains* analyzed. The synthesized compounds showed differential antibacterial activity, with compounds **1**, **2**, **7**, and **8** forming one group, and compounds **3**–**6** forming another, based on their efficacy profiles. Among these, compounds **1**–**4** exhibited the highest activity, comparable to those of clinically used antibiotics (Figure 1; see Figures S1 and S2 in the Supplementary Materials).

In particular, the strongest effects were observed in the *E. coli* R4 strain and in *S. aureus*, as reflected in both MIC and MBC values (Figure 2, Figure 3, Figure 4 and Figure 5). Statistical analyses confirmed that the differences in activity among the compounds tested were significant (*p* < 0.05; Table 1). These findings are of particular relevance in the context of combating multidrug-resistant pathogens among both Gram-positive and Gram-negative bacteria.

#### 2.1.3. Cytotoxicity of Coumarin-Based Peptidomimetics **1**–**8**

**Figure 6:**
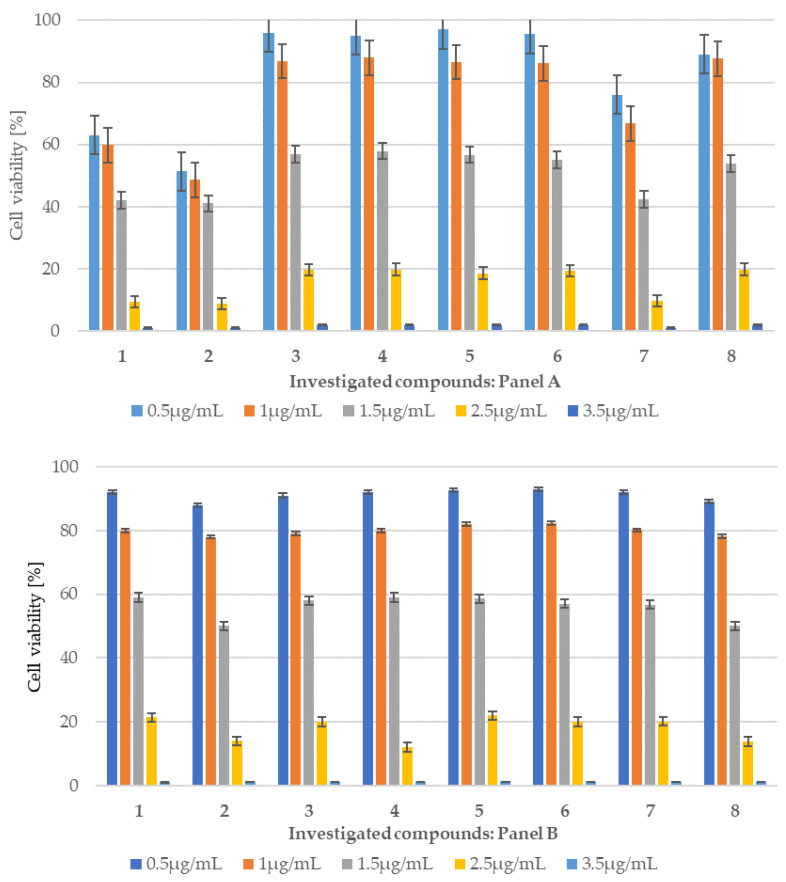
(BALB/c-3T3) (**panel A**) and (αT3-1) (**panel B**) in the MTT analysis after 24 h of incubation with 8 compounds. The *x*-axis features compounds **1**–**8**. Change in the level of peptidomimetics **1**–**8** over time. Different concentrations of each peptidomimetic are plotted separately in each panel.

Based on the MIC and MBC tests conducted in the bacterial strains analyzed and the selected antibiotics, an additional MTT test was performed to determine the cytotoxicity of the compound using the BALB/c-3T3 mouse embryonic fibroblast cell line and gonadotroph cell lines αT3-1 (Figure 6 panel A and B). When using culture as a model for healthy cells under physiological conditions to obtain results highly correlated with those obtained in vivo, it is necessary to choose a cell line with genotypic and phenotypic characteristics as close as possible to those of normal cells. The choice of the appropriate culture is largely determined by its origin and purpose, according to the guidelines of the European Collection of Cell Cultures (ECACC) (Figure S6 in the Supplementary Materials). BALB/c-3T3 mouse embryonic fibroblast cells and gonadotroph cell lines such αT3-1 were treated with the tested peptidomimetics from 1 to 8 at five different concentrations ranging from 0.5 to 3.5 μg/mL and incubated for 24 h. All peptidomimetics tested were not cytotoxic to BALB/c-3T3 cells and gonadotroph cell lines such αT3-1 in the analysis. Compounds **3** to **6** show the lowest cytotoxicity at a concentration of 0.5 µg/mL concentration, with viability percentages remaining above 96% (Figure 6 panel A and B). It should be noted that for compound **8** we observe slight differences in cell viability when using therapeutic concentrations (0.5 and 1 µg/mL). The tested concentration was 1 μg/mL, with viability percentages remaining above 99.50%.

However, a gradual reduction in viability was caused by compounds tested **1**–**8** at 1 μg/mL, with cell viability percentages ranging from 99.8% to 98% for compounds **1**–**4** and 52.50% for peptidomimetic **8**, (Figure 6, Panel A and B, Figure S6 in the Supplementary Materials). The obtained results were used to calculate the half-maximal inhibitory concentration (IC_50_) for peptidomimetics **2** (IC_50_ 6.58 µg/mL) and **8** (IC_50_ 1.71 µg/mL) which penetrated the blood–brain barrier to the greatest extent, after 24 h of incubation with the most active antimicrobial peptidomimetics in both cases of used cell lines. The obtained IC_50_ value is comparable to the concentration value achieved in the brain, which would suggest an increase or decrease in the therapeutic dose (own observations).

**Figure 7:**
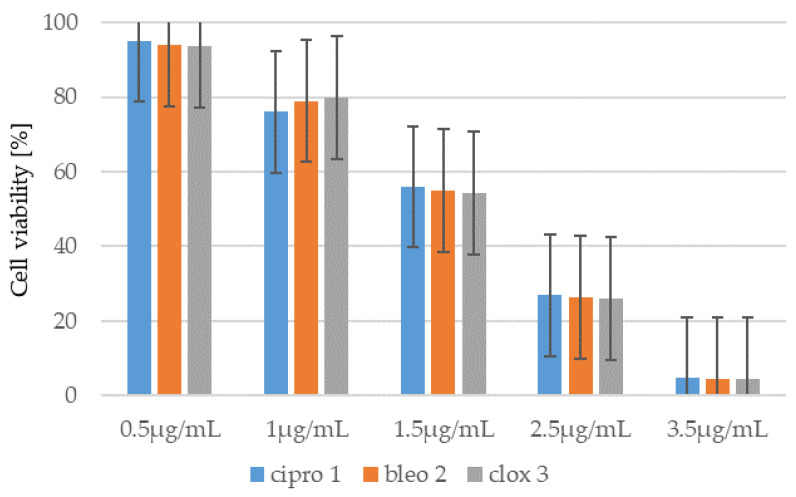
Example of measurement of cell viability (%) in the mouse embryonic fibroblast cell line (BALB/c-3T3) after 24 h of antibiotic incubation. The *x*-axis shows the antibiotics (ciprofloxacin (cipro), bleomycin (bleo), and cloxacillin (clox)) at different concentrations.

Similarly, to the compounds tested **1**–**8**, the MTT test was performed using representative antibiotics: ciprofloxacin (cipro), bleomycin (bleo) and cloxacillin (clox)) (Figure 7, and Supplementary Materials Figure S2). Similar concentrations of these antibiotics were used in the studies. The results obtained indicate that the cytotoxicity of the tested peptidomimetics **1**–**8** is lower than or comparable to that observed for these widely used drugs.

Antibiotic resistance among microorganisms represents a major global health concern that affects both humans and animals. Many pathogenic bacteria exhibit remarkable adaptability to various environmental conditions, allowing them to develop resistance to conventional antibiotics. In particular, incorporation of a CF_3_- substituent into the coumarin scaffold was associated with enhanced antimicrobial activity. A pronounced effect of CF_3_- substitution on the coumarin core was observed against the pathogenic strain *S. aureus* (ATCC 23235) as well as against the Gram-negative strains *E. coli* K12 (ATCC 25404), R2 (ATCC 39544), R3 (ATCC 11775), and R4 (ATCC 39543). Furthermore, the present findings suggest that the observed bactericidal activity of the tested compounds may be attributable to alterations in the spatial organization of bacterial cell membranes, ultimately leading to cell death [31].

### 2.2. In Vivo Experiment—Transfer of Coumarins to the CSF in Sheep

The next step of the study was to analyze the concentrations of the 8 compounds tested in cerebrospinal fluid (CSF) samples collected from the third ventricle of the brain continuously.

The sheep was the first animal to be domesticated by humans for agricultural purposes. Sheep have symptoms similar to those seen in humans with central nervous system (CNS) disease [57]. The entry of drugs into the large human brain is a particular problem for therapies such as ‘gene silencing’, which require drug delivery to the affected parts of the brain. Sheep have large brains, shaped similarly to the human brain, and are excellent models for studying CNS disease. Sheep can perform cognitive tasks, which is useful in reducing abnormalities in patients because they are based on similar brain areas and neural mechanisms.

#### 2.2.1. CSF Coumarins Concentration

The development of novel, non-toxic coumarin-based compounds that selectively exert toxicity toward bacterial cells is of considerable importance for antibacterial therapy, particularly in the context of complex infections affecting the central nervous system (CNS).

**Figure 8:**
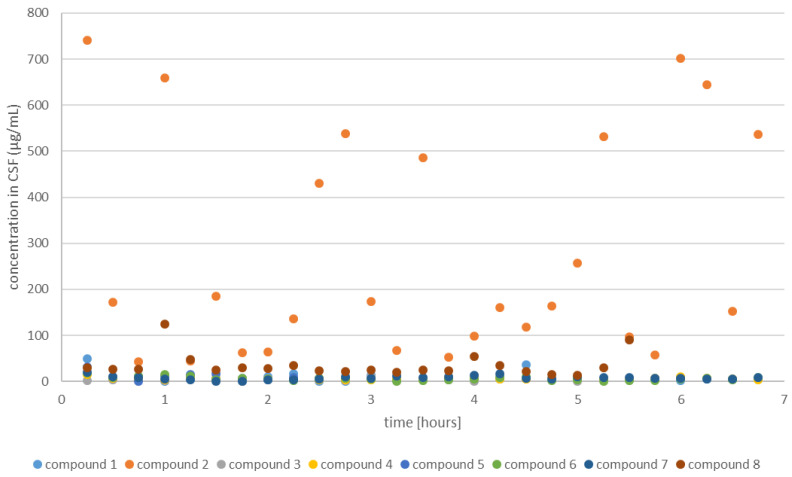
Time-dependent concentration profile of the investigated coumarin derivative in sheep cerebrospinal fluid (CSF).

Conducted experiments revealed that the transfer to the CSF (coumarin concentration level higher than 0) was statistically confirmed for 6 coumarins before centrifugation (**2**–**6**) and **8** after centrifugation (**2**, **7**, and **8**). On average, the coumarin concentration was the highest for a compound **2** (355.85 before and 118.32 µg/mL after centrifugation) (Figure 8, orange squares). In addition, mass spectrometric analysis of cerebrospinal fluid (CSF) was performed following the administration of compound **2**. The spectrum, acquired using electrospray ionization (ESI) in positive ion mode, revealed the presence of the compound as its sodiated ion (*m*/*z* 471) in the CSF (Figure S5, Supporting Information). Based on the absorbance analysis of three peptidomimetics (**1**, **2**, **7**) before and after centrifugation, we found statistically significant results only for one coumarin (adjusted *p*-value = 1.18 × 10^−5^ in both cases).

It was also observed that compound **8** is capable of crossing the blood–brain barrier; however, its concentration in the cerebrospinal fluid (CSF) is significantly lower than that of compound **2.** This suggests that the observed phenomenon is influenced not only by the presence of the trifluoromethyl (–CF_3_) group but also by the overall molecular structure.

The CF_3_ group is highly lipophilic and electronegative, meaning its addition often increases a molecule’s lipophilicity, reducing polarity and enhancing partitioning into lipid membranes, facilitating passive diffusion across the BBB. Because passive (non-saturable) diffusion favors small, lipophilic compounds, CF_3_ modifications are frequently used to tune drug-like molecules toward CNS permeability. C-F bonds are strong and metabolically stable: introducing CF_3_ groups typically increases resistance to metabolic degradation, thus prolonging systemic circulation and improving the chances of reaching brain tissue. Additionally, CF_3_ can influence molecular conformation via inductive and steric effects, optimizing receptor binding—including for those expressed in the CNS. Retrospective analyses indicate that while CF_3_ substitution does not universally improve pharmacological activity, specific targets and scaffolds do benefit from CF_3_, especially in CNS-active compounds. For example, a glucocorticoid receptor ligand lost agonist activity when CF_3_ was replaced by bulkier alkyl groups, underscoring the unique role of CF_3_ in aligning structure and function. Numerous FDA-approved drugs (about 19 studied) include CF_3_ moieties, underscoring its widespread value in drug development. BBB permeability is often characterized by a compound’s log BB (brain-to-blood concentration ratio) and log PS (permeability–surface area product). CF_3_ increases lipophilicity and often improves these parameters when other molecular properties (like molecular weight and hydrogen bonding) are controlled. Adding CF_3_ can improve log BB and log PS but must be balanced against size and PSA (polar Surface area), as excessive size or polarity impairs diffusion [54,55,56,57,58,59].

#### 2.2.2. Centrifugation of CSF Decreases the Coumarin Concentration

To check whether centrifugation of the CSF affects coumarin levels after entering the brain, we paired the data values when they were available (**1**, **2**, **7**, **8**). For coumarins **2** and **8** we observed a significant decrease in concentration level after centrifugation (Table 2). Plasma substances such as gases (O_2_, CO_2_), lipid-soluble compounds (ethanol, ether, steroid hormones, thyroid hormones and some lipophilic drugs) or peptides of 400–800 Da reach the internal environment of the brain by simple diffusion. Other routes of penetration of molecules into the central nervous system are selectively controlled by the activity of the BBB [12,60]. It is generally accepted that the rate at which substances penetrate brain tissue is inversely proportional to the size of the molecules and directly proportional to their solubility in lipids. Therefore, polarized, hydrophilic compounds pass more slowly. Some substances penetrate the barrier very slowly, while their related compounds pass much faster. This is the case for dopamine and serotonin, whose penetration into the brain is very limited, while their precursors (L-dopa and 5-hydroxytryptophan) break it much more easily [17,21,58,59,60]. The same is true for selected antibiotics such as cycloserine and chloramphenicol. This means that their concentration in the brain is lower than in the blood, and this may reduce the effectiveness of treating infections of the central nervous system, but their concentration in the brain may still be lower than in other tissues [25,26].

#### 2.2.3. Change in Coumarin Concentration Levels over Time

Time-resolved spectrophotometric measurements were performed to determine the concentrations of the investigated compounds in CSF. A pronounced influence of the structural features of the examined coumarin derivative on the analyte concentration within the studied temporal regime was observed (adjusted *p*-value = 0.024) (Figure 8).

Due to the existence of the barrier, changes in the osmolarity of the brain’s extracellular fluid and cerebrospinal fluid exhibit a significant time delay in relation to changes in the osmolarity of other body fluids. These unique features are essential for maintaining effective homeostasis within the central nervous system [16,17,18,19,20,21,22,23,24,25,46,60,61]. The cells of the blood–brain barrier capture and inactivate many substances from the blood, but are not capable of pinocytosis, which in other capillaries is the main route of passage of organic compounds of high molecular weight. Brain capillary cells contain numerous mitochondria and exhibit high metabolic activity. This activity provides the necessary energy for active transport across the lipid bilayer using protein carriers. This process is regulated by several tight junction proteins and adhesion molecules. To our current knowledge, the second mechanism relies on a large number of influx and efflux transporters and other membrane proteins, including members of G protein-coupled receptors and peptidoglycan [37,38,39,40,41,42,43,44,45,46,47,48,49,50,51,52,53,54,55,56,57,58,59,60,61,62,63,64,65,66,67]. Their ability to bind to a wide variety of ligands and diverse signaling profiles position them as ideal candidates for drug-targeted therapies.

The presented study showed that our compounds, especially those with trifluoromethylcoumarins in the fourth position, had a good ability to cross the BBB/BCSFB in sheep. After analyzing the CSF, we found that 25% of the attached coumarins with the CF_3_ group was in the free form and 75% in the form bound to receptor proteins. The coumarin derivative designated compound **2** is particularly notable, as its structure can determine the crossing of the BBB/BCSFB through interactions with the receptors of many proteins. Coumarin **2** does not dissolve in water but binds well to proteins, which we observed very clearly in the supernatant before and after centrifugation without coumarin and with coumarin. The activity of compound **2** is most likely attributable to the ability of membrane receptors to recognize specific structural motifs, such as the ester group, which can undergo modifications at its stereogenic center (see the Supplementary Material, Figures S3 and S4). It is also possible that coumarin **2** overcomes this BBB/BSCFB by binding most of the other coumarins analyzed without the CF_3_ group to stereospecific receptors. Furthermore, coumarin compounds **3**, **5**, and **7** crossed the BBB/BCSFB, but there they were partially metabolized, as we can see from the regression and absorbance curves in the form of points above and below the curve and analysis in MIC and MBC tests of their antimicrobial activity.

Demonstrating antibacterial activity, as well as the transfer of the newly designed coumarin derivatives to the CSF of sheep, may provide the basis for the therapeutic application of these compounds not only in the tested animal species or the entire group of ruminants, but also in humans suffering from neurological disorders caused by CNS infections and/or neurodegenerative diseases [7,9]. Since the integrity of BBB/BCSFB is altered during systemic inflammation or infection, allowing many unwanted substances to gain access to the brain [24,27,28,51], we plan to continue research on the direct penetration of new coumarin derivatives directly in the CNS.

## 3. Materials and Methods

### 3.1. Chemistry of Newly Synthesized Coumarin Derivatives

All reagents and solvents were purchased from Sigma-Aldrich (Saint Louis, MI, USA). All solvents were of analytical grade and were used without prior distillation. Merck silica gel 60 F254 plates were used for TLC (Thin-Layer Chromatography) analysis. The crude reaction mixtures were purified using column chromatography on Merck silica gel 60/230–400 mesh (Rahway, NJ, USA), with an appropriate mixture of hexanes and ethyl acetate as eluent. Nuclear magnetic resonance (NMR) spectra were performed on a Varian apparatus (Varian, Saint Louis, MI, USA) (400 MHz) and (500 MHz); the mass spectrometer was from Waters Company, Milford, MA, USA. Chemical shifts are expressed in ppm and the coupling constant (J) is expressed in Hz using TMS as an internal standard. High-resolution mass spectra were acquired on a Maldi SYNAPT G2-S HDMS apparatus (Waters) with a QqTOF analyzer. To prove the ability of the established protocol, each reaction was repeated at least three times. Cerebrospinal fluid (CSF) samples were analyzed for the presence of the investigated coumarin derivative **2** using a SCIEX API 3000 triple quadrupole mass spectrometer (Science, Engineering, and Technology (SCIEX, Concord, ON, Canada)) equipped with an electrospray ionization (ESI) source operated in positive ion mode.

### 3.2. General Method for Imine Preparation

A mixture of 7-amino-4-methylcoumarin (1.5 g, 0085 mmol) and substituted aromatic aldehyde (0.017 mmol) was refluxed in 25 ml of absolute alcohol and 0.5 mL of acetic anhydride for 6 h, then, the solvent was removed under reduced pressure. The resulting crude Schiff base was washed with cold water and recrystallized by using appropriate solvents. The purity of the compounds was confirmed by TLC using silica gel as the stationary phase, ethyl acetate: cyclohexane (1:2) as the mobile phase and melting point; compounds **3**, **6** and **8** were previously synthesized [29,30].

### 3.3. General Procedure for Synthesis of 4U-MCR Compounds

To the solution of the corresponding amine (1 equiv.) in methanol (1 mL), the respective aldehyde (1 equiv.) was added and stirred at room temperature for 30 min, followed by the addition of carboxylic acid (1 equiv.), and then the mixture for another 15 min. Then isocyanide (1 equiv.) was added to the reaction mixture and stirred overnight at room temperature. The solvent was evaporated off under reduced pressure, and column chromatography was performed to obtain pure compounds.

### 3.4. General Procedure for Synthesis of 3U-MCR Compounds

To the solution of the corresponding amine (1 mmol, 1 equiv.) in methanol (1 mL), the respective aldehyde (1 equiv.) was added and stirred at room temperature, for 30 min, followed by the addition of citric acid (1 equiv.), and then the mixture was stirred for another 15 min. Then isocyanide (1 equiv.) was added to the reaction mixture and stirred overnight at room temperature. The solvent was evaporated off under reduced pressure, and column chromatography was performed to obtain pure compounds.

### 3.5. Compound ***1***

^1^H NMR (400 MHz, CDCl3) δ 7.42 (d, J = 8.3 Hz, 1H), 7.11 (d, J = 8.7 Hz, 2H), 7.01 (d, J = 8.7 Hz, 2H), 6.81–6.74 (m, 2H), 6.68–6.61 (m, 2H), 6.22 (d, J = 1.4 Hz, 2H), 6.08 (s, 1H), 4.37 (d, J = 5.7 Hz, 2H), 3.73 (s, 3H), 3.68 (s, 3H), 2.37 (s, 3H), 2.30 (dt, J = 6.9, 3.4 Hz, 2H), 2.17–2.08 (m, 2H), 1.93–1.84 (m, 2H); ^13^C NMR (100 MHz, CDCl3) δ 177.3, 172.5, 169.7, 160.3, 159.6, 158.9, 151.7, 142.8, 131.5, 129.9, 128.9, 125.6, 124.8, 119.6, 119.2, 115.5, 113.9, 65.8, 64.4, 55.2, 55.1, 43.2, 33.8, 32.8, 20.2, 18.5. HRMS (ESI) [M + Na] + *m*/*z* calcd. for C_32_H_32_N_2_O_8_Na 595.2056; found 595.2049; Elemental anal. calcd. for C_32_H_32_N_2_O_8_; C: 67.12%; H: 5.63%; N: 4.89%; found 67.08%; H: 5.58%; N: 4.81%.

### 3.6. Compound ***2***

^1^H NMR (400 MHz, CDCl3) δ 7.50–7.43 (m, 2H), 7.43–7.26 (m, 4H), 6.83–6.71 (m, 1H), 6.61 (dd, J = 8.9, 2.4 Hz, 1H), 6.40–6.31 (m, 2H), 6.08 (d, J = 4.7 Hz, 1H), 5.00 (d, J = 4.7 Hz, 1H), 4.18 (q, J = 7.2 Hz, 2H), 4.10–3.86 (m, 2H), 1.22 (t, J = 7.2 Hz, 3H); ^13^C NMR (100 MHz, CDCl3) δ 170.1, 169.3, 160.1, 156.6, 150.3, 137.2, 129.5, 129.0, 127.1, 112.3, 104.4, 99.3, 61.7, 61.2, 41.7, 14.0. HRMS (ESI) [M + Na] + *m*/*z* calcd. for C_22_H_19_F_3_N_2_O_5_Na 471.1144; found 471.1138; Elemental anal. calcd. for C_22_H_19_F_3_N_2_O_5_; C:58.93%; H: 4.27%; N: 6.25%; found C: 58.88%; H: 4.21%; N: 6.19%.

### 3.7. Compound ***4***

^1^H NMR (400 MHz, CDCl_3_) δ 7.41 (dd, J = 8.9, 2.0 Hz, 1H), 7.36 (d, J = 8.7 Hz, 2H), 6.90 (d, J = 8.7 Hz, 2H), 6.61 (dd, J = 8.9, 2.3 Hz, 1H), 6.51 (t, J = 5.4 Hz, 1H), 6.41–6.33 (m, 2H), 5.97 (d, J = 4.2 Hz, 1H), 4.91 (d, J = 4.2 Hz, 1H), 4.16 (q, J = 7.1 Hz, 2H), 4.13–3.87 (m, 2H), 3.77 (s, 3H), 1.23 (t, J = 7.1 Hz, 3H); ^13^C NMR (100 MHz, CDCl_3_) δ 170.2, 169.2, 160.1, 156.6, 150.3, 129.0, 128.3, 115.0, 112.4, 109.3, 104.5, 99.4, 61.7, 60.7, 55.3, 41.6, 14.0. HRMS (ESI) [M + Na] + *m*/*z* calcd. for C_23_H_21_F_3_N_2_O_6_Na 501.1249; found 501.1242; Elemental anal. calcd. for C_23_H_21_F_3_N_2_O_6_; C: 57.74%; H: 4.42%; N: 5.86%; found C: 57.74%; H: 4.42%; N: 5.86%.

### 3.8. Compound ***5***

^1^H NMR (400 MHz, CDCl_3_) δ 7.44–7.29 (m, 3H), 7.24–7.13 (m, 8H), 7.07–7.01 (m, 2H), 6.29–6.20 (m, 3H), 4.16 (q, J = 7.1 Hz, 2H), 4.05 (dd, J = 9.0, 5.3 Hz, 2H), 3.52–3.40 (m, 2H), 2.38 (s, 3H), 1.26 (t, J = 7.1 Hz, 3H); ^13^C NMR (100 MHz, CDCl3) δ 171.2, 169.4, 160.1, 151.5, 142.7, 134.5, 133.4, 130.3, 129.0, 128.9, 128.6, 128.4, 126.8, 124.6, 119.4, 115.7, 77.3, 77.0, 76.7, 64.8, 61.5, 41.7, 41.6, 18.5, 14.1. HRMS (ESI) [M + Na]+ *m*/*z* calcd. for C_31_H_30_N_2_O_7_Na 565.1951; found 565.1948; Elemental anal. calcd. for C_31_H_30_N_2_O_7_; C: 68.62%; H: 5.57%; N: 5.16%; found C: 68.53%; H: 5.49%; N: 5.09%.

### 3.9. Compound ***7***

^1^H NMR (400 MHz, CDCl_3_) δ 7.59–7.51 (m, 2H), 7.43 (dd, J = 8.9, 1.9 Hz, 1H), 7.40–7.31 (m, 2H), 6.60 (dd, J = 8.9, 2.4 Hz, 1H), 6.54 (t, J = 5.2 Hz, 1H), 6.43–6.32 (m, 2H), 5.97 (d, J = 4.5 Hz, 1H), 4.95 (d, J = 4.5 Hz, 1H), 4.18 (q, J = 7.1 Hz, 2H), 4.14–3.86 (m, 2H), 1.25 (t, J = 7.2 Hz, 3H); ^13^C NMR (100 MHz, CDCl3) δ 169.2, 156.6, 149.8, 136.3, 132.7, 128.7, 123.2, 112.3, 104.8, 99.4, 61.8, 60.6, 41.7, 14.0. HRMS (ESI) [M + Na] + *m*/*z* calcd. for C_22_H1_8_BrF_3_N_2_O_5_Na 549.0249; found 549.0243; Elemental anal. calcd. for C_22_H_18_BrF_3_N_2_O_5_; C:50.11%; H:3.44%; N:5.31%; found C:50.03%; H:3.38%; N:5.24%.

### 3.10. Microorganisms and Media—In Vitro Experiment Number 1-Bactericidal Effects

The antimicrobial activity of synthesized coumarin-based peptidomimetics was tested on bacterial strains known to have a potential pathogenic effect on the animal and/or human body. *Staphylococcus aureus*, *Escherichia coli* K-12 and R2–R4 strains were received from Prof. Jolanta Łukasiewicz at the Ludwik Hirszfeld Institute of Immunology and Experimental Therapy (Polish Academy of Sciences, Warsaw, Poland). The referenced bacterial strains of *E.coli* (K-12 ATCC 25404, R2 ATCC 39544, R3 ATCC 11775, and R4 ATCC 39543) and *S. aureus* (ATCC 23235) were provided by collection of LGC Standards U.K. or ATCC and used according to the literature recommendation [31,49]. The bacteria were grown in a tryptic soy broth (TSB; Sigma-Aldrich, Saint Louis, MI, USA) and on agar plates containing TSB medium at 25 °C. Alternatively, TSB agar plates were used. The specific growth rate (μ) according to first-order kinetics was measured using a microplate reader (Thermo, Multiskan FC, Vantaa, Finland) at 605 nm in TSB medium and used for the MIC and MBC tests, as described in detail in previous work [31,49].

Our studies using sheep brain focus on the use of peptidomimetics (Figure 1), [24,54] with antimicrobial activity, which effectively penetrate the blood–brain barrier (BBB). Antimicrobial resistance is one of the main problems in healthcare. Therefore, such an approach is known for brain-penetrating peptides in the aspect of human diseases caused by pathogenic bacteria *E. coli* and *S. areus*, which induce other diseases in mammalian organisms. This is because *E. coli* or *S. aureus* LPS bacteria can cause sepsis, leading to renal medullary perfusion in the fetal sheep brain and renal cortex hypoxia, causing brain dysfunction and lethargy in premature and adult Merino sheep [15,22,27,28,29,49,50,62,63,64,65,66,67,68,69].

### 3.11. The Determination of the Minimum Inhibitory Concentration (MIC) and the Minimum Bactericidal Concentration (MBC)

MIC was estimated using a microtiter plate method using 48- or 96-well plates [26,27,34,35,36,53]. First, the precursor and TIL solutions were prepared in DMSO at 20 mg mL−1. Fifty microliters of each solution were placed in the first row of the plate. Next, 25 μL of sterile TSB medium was added to the other wells, and serial dilutions were performed. Then 200 μL of inoculated TSB medium containing resazurin (0.02 mg/mL) was added as an indicator to all wells. TSB medium was inoculated with 10(6) colony-forming units (CFU) mL−1 (approximately 0.5 McFarland units) of the bacterial strains. The plates were incubated at 30 °C for 24 h. The color changes from blue to pink or yellowish with turbidity were taken as positive, and the lowest concentration at which there was no visible color change was the MIC. The MBC was estimated based on the measurement of dehydrogenase activity in cultures after 24 h incubation without ILs. Four milliliters of a dense culture (approximately 10(9) CFU mL−1) that was incubated for 24 h in TSB medium at 25 °C were added to identical test tubes. The tested compounds were added to the test tubes until the mixture reached final concentrations of 1–25 mg mL^−1^. The cultures containing the TILs were then incubated for 1 h at 30 °C. Next, 0.1 g of CaCO_3_ and 0.1 mL of a 3% triphenyltetrazolium chloride (TTC) solution were added to the test tubes. The test tubes were sealed with parafilm and incubated for 1 h at 30 °C in darkness. The lowest concentration at which there was no visible red color (formazan) was taken as the MBC.

A similar MIC and MBC test design was applied to CSF as a control for antimicrobial properties as well as to the peptidomimetics used (see Supplementary Materials Figure S3).

### 3.12. Experiment No. 2—MTT Assay

The cytotoxic effects of the tested peptidomimetics **1**–**8** on BALB/c-3T3 mouse fibroblast cells and gonadotroph cell lines such αT3-1 came from the ATCC collection.

The Balb/c3T3 cell line (ATCC) has been described in [70,71]. The αT3-1 cell line has been described in [72,73,74]. All cells were cultured in Petri dishes in DMEM in a 5% CO_2_ incubator. Balb/c3T3 cells required supplementation with 10% fetal bovine serum (PAA Laboratories, cat. no. A15-751), whereas αT3-1 cells were maintained in monolayer cultures in DMEM supplemented with 10% fetal bovine serum, 100 U/ml penicillin G, and 0.1 mg/mL streptomycin (Invitrogen, China) in humidified 5% CO_2_, 95% air, at 37 °C. The cells were passed at ~95% confluence using a trypsin-EDTA solution (0.05% trypsin, 0.5 mM EDTA). Cells were kept in phenol red-free medium containing charcoal-treated fetal bovine serum for 4 days before the experiments. All cell lines were periodically treated with plasmacin (Invivogen #ant-mpt-1, 10 μg/mL for 2 weeks) and examined for mycoplasma contamination by DAPI staining or PCR as described in [72,73,74,75].

Both cell lines were determined using the MTT assay after 24 h of incubation at five concentrations. The MTT test is based on the ability of mitochondrial dehydrogenase enzymes to convert an orange, water-soluble tetrazolium salt (3-(4,5-dimethylthiazol-2-yl)-2,5-diphenyltetrazolium bromide) into an insoluble formazan, which is a dark blue product of the above reaction. After the formazan crystals were placed in DMSO or isopropanol, a colored solution formed, and the intensity was measured spectrophotometrically within the wavelength range of 492–570 nm. The amount of colored reduced MTT was proportional to the oxidative activity of the cell mitochondria and, under strictly defined experimental conditions, to the number of metabolically active (living) cells in the population. The MTT test can also be used to determine cell viability in populations of cells that no longer divide, but are metabolically active. The MTT test is currently the most widely used to assess cytotoxic activity and is recommended as a reference by international standards-setting organizations [70,71,72,73,74,75,76,77,78,79,80,81,82,83,84].

### 3.13. Experiment No. 3—Transfer of Coumarins to the CSF in Sheep In Vivo-Animal Management

Four mature Polish Merino sheep (aged 10 months), weighing 50 ± 1 kg, were used in the experiment. They were raised indoors at the Kielanowski Institute of Animal Physiology and Nutrition, Polish Academy of Sciences (Jabłonna near Warsaw, Poland) under natural light conditions (52° N, 21° E). The animals were fed twice a day according to their physiological status. Animal nutrition was based on pellet concentrate and hay according to the recommendations of the National Research Institute of Animal Production (Krakow–Balice, Poland) and the National Institute of Agricultural Research (France) [28]. During the experimental period, the sheep were kept in individual pens with free access to water and mineral licks, as well as visual, olfactory and tactile contact with individuals of the same species.

### 3.14. Third Ventricle (IIIv) Cannulation

One month before the experiment (September), the sheep were subjected to surgical implantation of a cannula into the IIIv (outer diameter 1.2 mm, frontal 31.0 mm), according to the stereotaxic coordinate system for the hypothalamus of the sheep in the procedure described by [49,50] and by Traczyk and Przekop [69]. Implantation was performed under general anesthesia (xylazine: 40 mg/kg of body mass, intravenously; xylapan and ketamine: 10 to 20 mg/kg of body mass, intravenously; Bioketan, Vetoquinol Biowet, Puławy, Poland) through a hole drilled in the skull. A guide cannula was attached to the skull with stainless steel screws and dental cement. The external opening of the canal was closed with a stainless-steel cap. After surgery, the ewes were injected for 4 days with antibiotics (1 g streptomycin and 1,200,000 IU of benzylpenicillin; Polfa, Poland) and with analgesics (metamizole sodium 50 mg/animal; Biovetalgin, Biowet Drwalew, Poland or meloxicam 1.5 mg/animal; Metacam, Boehringer Ingelheim, Germany). The placement of the cannula in IIIv was confirmed by the outflow of CSF during surgery and visually after slaughter.

### 3.15. Drug Preparation

The day before animal administration, tested coumarins (synthesized at the Institute of Organic Chemistry) were dissolved in a dimethyl sulfoxide (DMSO, Chempur, Piekary Śląskie, Poland), at a concentration of 1 mg/kg body weight. After 24 h, the coumarin solution was diluted in physiological saline in a 1:10 ratio (50 mg/500 mL) for intravenous (iv) infusion.

### 3.16. Experimental Design and Sample Collection

Estrus synchronization was performed in mid-October after the convalescence period, when the sheep reached full sexual maturity, showing complete estrous cycles. Polyester polyurethane sponges (Chronogest–CR) containing 20 mg of flugestone acetate (Intervet, Boxmeer, The Netherlands) were inserted into the sheep intravaginally for 14 days and then removed at the time of injection of pregnant mare’s serum gonadotropin (PMSG) injection (500 iu, Folligon, Intervet, Boxmeer, The Netherlands). Estrus occurred within 24 to 48 h after PMSG administration and its duration was additionally controlled using a vasectomized ram. A continuous collection of IIIv CSF samples was performed in every sheep twice on day 10 of consecutive cycles (luteal phase), beginning with the first synchronized estrus. The CSF flowed out at a flow rate of 20 μL/min, which is within the range of the CSF turnover rate in the sheep’s CNS [41,42]. The total time for the CSF sampling was 7 h (from 9:00 to 16:00), allowing the collection of 28 samples (approximately 300 μL/15 min) from each sheep. The CSF samples were collected in Eppendorf tubes placed on ice, using calibrated 1.0–mL gas-tight syringes and a CMA/402 microinjection pump (CMA, Stockholm, Sweden). The coumarin solution in saline (50 mg/500 mL) was administered intravenously, as a drip, over 15 min, through a catheter inserted into the jugular vein. Coumarin administration was started after collecting at least one ‘clear’ control CSF samples (between 9:30 and 10:00). During the collection and treatments, the sheep were kept in pairs in the experimental room in comfortable cages, where they could lay and to which they had previously been adapted for at least 2 days.

### 3.17. Measurement of Coumarin Concentration in the CSF of Sheep

Coumarins were quantified in the CSF samples immediately after filling the tubes using a spectrophotometric method. Specifically, CSF samples were centrifuged at 10,000× *g* for 60 s at 4 °C and the amounts of 130 μL of the supernatant were placed on a 96-well microplate. Data acquisition was performed using a SpectraMax iD3 multimode microplate reader (Molecular Devices, San Jose, CA, USA) at the absorption maximum of the compound to ensure optimal sensitivity and accuracy. The absorbance was read against the Ringer-Locke solution as a blank. For each compound the wavelength was determined based on the results of a preliminary scanning of absorbance in a 230–1000 nm spectrum with 10 nm intervals. The concentrations of the coumarin derivatives in the CSF were calculated from the standard curves prepared for each compound.

### 3.18. Mass Spectrometric Determination of Coumarin 2 in Cerebrospinal Fluid (CSF) Collected from Sheep

Sheep CSF samples were collected and stored at −80 °C until further analysis. For protein precipitation, 100 µL of sample was mixed with 300 µL of ice-cold acetonitrile/water solution (80:20, *v*/*v*) containing 0.1% acetic acid. The mixture was vortexed for 1 min and incubated on ice for 30 min to facilitate protein precipitation and reduce matrix interferences. The samples were centrifuged at 14,000× *g* for 15 min at 4 °C and the clear supernatant was collected for mass spectrometry analysis. The samples were analyzed using a SCIEX API 3000 triple quadrupole mass spectrometer equipped with an electrospray ionization (ESI) source operating in positive ion mode. The ESI source parameters were set as follows: capillary voltage, 4.5 kV; nebulizer gas temperature, 200 °C; nebulizer pressure of 4.0 bar; curtain gas pressure, 35 psi. The samples were infused at a flow rate of 10 µL/min using a syringe pump. Mass spectra were acquired in full-scan mode over an *m*/*z* range of 500 to 2000.

### 3.19. Statistical Analysis

The Shapiro–Wilk test was used to check the normality of the data. Depending on the distribution of the data, two types of tests were used in the further analysis: the parametric Student’s *t* test was used for samples with normal distribution, while the nonparametric Wilcoxon test was used for samples with nonnormal distribution. A linear regression model was used to check whether there is a significant increasing or decreasing trend of the coumarin level (or absorbance) over time. In all tests, a 5% significance level was used. The estimated *p*-values were corrected for multiple tests using the Benjamini–Hochberg method.

## 4. Conclusions

The present study demonstrates that coumarin-based antibacterial-activity peptidomimetics can constitute a valuable experimental approach to investigate the transfer of coumarin derivatives across the blood–brain and blood–cerebrospinal fluid barriers (BBB/BCSFB) using a sheep model. The findings provide evidence supporting the potential therapeutic application of newly synthesized coumarin derivatives in the prevention and treatment of infections of the central nervous system (CNS) caused by pathogenic bacteria in both veterinary and human medicine. The CF_3_ and Me groups attached to the coumarin scaffold demonstrated an improved effect on antimicrobial activity against Gram-positive bacteria *Staphylococcus aureus* (ATCC 23235), as well as Gram-negative bacteria, including various strains of *E. coli* (K12 (ATCC 25404), R2 (ATCC 39544), R3 (ATCC 11775) and R4 (ATCC 39543)). Among the compounds evaluated, derivatives containing a CF_3_ substituent exhibited superior penetration across brain barriers. Additionally, toxicity assays confirmed that the most promising derivatives, when tested via the MTT assay on neuronal and non-neuronal cell lines, did not exert harmful effects on nerve cells. Together, these results contribute to a better understanding of the mechanisms underlying the disruption of BBB and highlight the potential of coumarin derivatives as candidates for further therapeutic development. A key limitation of this study is the use of a single dose of coumarin derivatives, selected on the basis of antibacterial minimum inhibitory concentration (MIC) and MTT cytotoxicity assays. This restriction may limit the generalizability of the observed effects on brain barrier permeability. Therefore, future studies should explore a broader range of concentrations and dosing regimens to establish dose–response relationships. Moreover, the incorporation of strategies designed to enhance transcellular transport, such as the use of peptide-based carriers, or the application of alternative delivery methods that bypass the BBB (e.g., local drug administration), may further improve the translational relevance and therapeutic efficacy of these compounds.

## Figures and Tables

**Table 1:** Statistical analysis of all analyzed compounds by MIC, MBC, and MBC/MIC; * *p* < 0.05, ** *p* < 0.01.

No. of Samples	1, 2, 3, 4	5, 6, 7, 8	Type of Test
*E.coli* K12	**	*	MIC
*E.coli* R2	**	*	MIC
*E.coli* R3	**	*	MIC
*E.coli* R4	**	*	MIC
*E.coli* K12	*	**	MBC
*E.coli* R2	*	**	MBC
*E.coli* R3	*	**	MBC
*E.coli* R4	*	**	MBC
*E.coli* K12	**	*	MBC/MIC
*E.coli* R2	**	*	MBC/MIC
*E.coli* R3	**	*	MBC/MIC
*E.coli* R4	**	*	MBC/MIC
*Staphylococcus aureus*	*	*	MIC
*Staphylococcus aureus*	*	*	MBC
*Staphylococcus aureus*	**	**	MBC/MIC

**Table 2:** Average coumarin concentration values were observed before and after centrifugation for compounds **116** > **21** > **111**.

Compound	Value (μg/mL) Without Centrifugation	Mean (μg/mL) Value with Centrifugation
**1**	10 (+/−5)	2 (+/−5)
**2**	350 (+/−5)	2 (+/−5)
**3**	2.5 (+/−5)	nd
**4**	7 (+/−5)	nd
**5**	5 (+/−5)	nd
**6**	8 (+/−5)	nd
**7**	6 (+/−5)	6 (+/−5)
**8**	30 (+/−5)	20 (+/−5)

## Data Availability

Data may be made available upon request.

## Supplementary Materials

The following supporting information can be downloaded at: https://www.mdpi.com/article/10.3390/ijms26199655/s1.

## Abbreviations

BBB—blood–brain barrier; BCSFB—blood-cerebrospinal barrier; CNS—central nervous system; CSF—cerebrospinal function; MIC—minimum inhibitory concentration; MBC—minimum bactericidal concentration; MTT-3-(4,5-dimethylthiazol-2-yl)-2,5-diphenyltetrazolium bromide; NMR—nuclear magnetic resonance, Science, Engineering, and Technology (SCIEX)

## References

[1] Gong X., Wang N., Zhu H., Tang N., Wu K., Meng Q. (2023). Anti-NMDAR antibodies, the blood-brain barrier, and anti-NMDAR encephalitis. Front. Neurol..

[2] Zhang L., Nan X., Zhou D., Wang X., Zhu S., Li Q., Jia F., Zhu B., Si Y., Cao S. (2024). Japanese encephalitis virus NS1 and NS1’ protein disrupts the blood-brain barrier through macrophage migration inhibitory factor-mediated autophagy. J. Virol..

[3] Oevermann A., Di Palma S., Doherr M.G., Abril C., Zurbriggen A., Vandevelde V.M. (2010). Neuropathogenesis of naturally occurring encephalitis caused by Listeria monocytogenes in ruminants. Brain Pathol..

[4] Fecteau G., George L.W. (2004). Bacterial meningitis and encephalitis in ruminants. Vet. Clin. N. Am. Food Anim. Pract..

[5] Filioussis G., Petridou E., Karavanis E., Giadinis N.D., Xexaki A., Govaris A., Kritas S.K.J. (2013). An outbreak of caprine meningoencephalitis due to Escherichia coli O157:H7. J. Vet. Diagn. Investig..

[6] Wang W., Cai M., Hu J., Zhang Z., Wang X., Chang X., Zhang F., Guo Ch Wang X. (2020). Mechanism of blood-brain barrier disruption by an Escherichia coli from lambs with severe diarrhea and meningoencephalitis. Microb. Pathog..

[7] Banks W.A. (2016). From blood–brain barrier to blood–brain interface: New opportunities for CNS drug delivery. Nat. Rev. Drug Discov..

[8] Peluffo H., Unzueta U., Negro-Demontel M.L. (2015). BBB-targeting, protein-based nanomedicines for drug and nucleic acid delivery to the CNS. Biotechnol. Adv..

[9] Abbott N.J. (2013). Blood–brain barrier structure and function and the challenges for CNS drug delivery. J. Inherit. Metab. Dis..

[10] Elmeliegy M.A., Carcaboso A.M., Tagen M., Bai F., Stewart C.F. (2011). Role of ATP-Binding Cassette and Solute Carrier Transporters in Erlotinib CNS Penetration and Intracellular Accumulation. Clin. Cancer Res..

[11] Pérez-Hernández M., Fernández-Valle M.E., Rubio-Araiz A., Vidal R., Gutiérrez-López M.D., O’Shea E., Colado M.I. (2017). 3,4-Methylenedioxymethamphetamine (MDMA, ecstasy) produces edema due to BBB disruption induced by MMP-9 activation in rat hippocampus. Neuropharmacology.

[12] Pandit R., Chen L., Götz J. (2020). The blood-brain barrier: Physiology and strategies for drug delivery. Adv. Drug Deliv. Rev..

[13] Ayrton A., Morgan P. (2001). Role of transport proteins in drug absorption, distribution and excretion. Xenobiotica.

[14] Pardridge W.M. (2002). Drug and gene targeting to the brain with molecular Trojan horses. Nat. Rev. Drug Discov..

[15] Yan E., Castillo-Meléndez M., Nicholls T., Hirst J., Walker D. (2004). Cerebrovascular responses in the fetal sheep brain to low-dose endotoxin. Pediatr. Res..

[16] Pardridge W.M. (2007). Blood-brain barrier delivery. Drug Discov. Today.

[17] Sakka A.L., Coll G., Chazal J. (2011). Anatomy and physiology of cerebrospinal fluid. Eur. Ann. Otorhinolaryngol. Head Neck Dis..

[18] Laterra J., Keep R., Betz L.A., Goldstein G.W., Siegel G.J., Agranoff B.W., Albers R.W., Fisher S.K., Uhler M.D. (1999). Blood-cerebrospinal fluid barrier. Basic Neurochemistry: Molecular, Cellular and Medical Aspects.

[19] Skipor J., Thiery J.C. (2008). The choroid plexus—Cerebrospinal fluid system: Undervaluated pathway of neuroendocrine signaling into the brain. Acta Neurobiol. Exp..

[20] Wang D., Guan S., Lu P., Li Y., Xu H. (2023). Extracellular vesicles: Critical bilateral communicators in periphery-brain crosstalk in central nervous system disorders. Biomed. Pharmacother..

[21] Goncalves A.R.A., De Felice F.G. (2021). The crosstalk between brain and periphery: Implications for brain health and disease. Neuropharmacology.

[22] Murray S.J., Mitchell N.L. (2022). The Translational Benefits of Sheep as Large Animal Models of Human Neurological Disorders. Front. Vet. Sci..

[23] He Y., Qu S., Wang J., He X., Lin W., Zhen H., Zhang X. (2012). Neuroprotective effects of osthole pretreatment against traumatic brain injury in rats. Brain Res..

[24] Galea I. (2021). The blood-brain barrier in systemic infection and inflammation. Cell. Mol. Immunol..

[25] McCallum N., Berger-Bachi B., Senn M.M. (2010). Regulation of antibiotic resistance in Staphylococcus aureus. Int. J. Med. Microbiol..

[26] Kadeřábková N., Mahmood A.J.S., Mavridou D.A.I. (2024). Antibiotic susceptibility testing using minimum inhibitory concentration (MIC) assays. Npj Antimicrob. Resist..

[27] Takahashi Y., Takahashi T., Usuda H., Carter S., Fee E.L., Furfaro L., Chemtob S., Olson D.M., Keelan J.A., Kallapur S. (2023). Pharmacological blockade of the interleukin-1 receptor suppressed Escherichia coli lipopolysaccharide-induced neuroinflammation in preterm fetal sheep. Am. J. Obstet. Gynecol. MFM..

[28] Matsushima S., Maeda K., Hayashi H., Debori Y., Schinkel A.H., Schuetz J.D., Kusuhara H., Sugiyama Y. (2008). Involvement of Multiple Efflux Transporters in Hepatic Disposition of Fexofenadine. Mol. Pharmacol..

[29] Patra A., Chen X., Sadowska G.B., Zhang J., Lim Y.-P., Padbury J.F., Banks W.A., Stonestreet B.S. (2017). Neutralizing anti-interleukin-1β antibodies reduce ischemia-related interleukin-1β transport across the blood-brain barrier in fetal sheep. Neuroscience.

[30] Chen X., Sadowska G.B., Zhang J., Kim J.E., Cummings E.E., Bodge C.A., Lim Y.P., Makeyev O., Besio W.G., Gaitanis J. (2015). Neutralizing anti-interleukin-1beta antibodies modulate fetal blood-brain barrier function after ischemia. Neurobiol. Dis..

[31] Peng X.M., Damu G.L.V., Zhou C.H. (2013). Current developments of coumarin compounds in medicinal chemistry. Curr. Pharm. Des..

[32] Feng D., Zhang A., Yang Y., Yang P. (2020). Coumarin-containing hybrids and their antibacterial activities. Arch. Pharm..

[33] Qin H.L., Zhang Z.W., Ravindar L., Rakesh K.P. (2020). Antibacterial activities with the structure-activity relationship of coumarin derivatives. Eur. J. Med. Chem..

[34] Kowalczyk P., Madej A., Paprocki D., Szymczak M., Ostaszewski R. (2020). Coumarin Derivatives as New Toxic Compounds to Selected K12, R1-R4 *E. coli* Strains. Materials.

[35] Kowalczyk P., Wilk M., Parul P., Szymczak M., Kramkowski K., Raj S., Skiba G., Sulejczak D., Kleczkowska P., Ostaszewski R. (2021). The Synthesis and Evaluation of Aminocoumarin Peptidomimetics as Cytotoxic Agents on Model Bacterial, *E. coli* Strains. Materials.

[36] Kowalczyk P., Koszelewski D., Brodzka A., Kramkowski K., Ostaszewski R. (2023). Evaluation of Antibacterial Activity against Nosocomial Pathogens of an Enzymatically Derived α-Aminophosphonates Possessing Coumarin Scaffold. Int. J. Mol. Sci..

[37] Kalkhambkar R.G., Kulkarni G.M., Kamanavalli C.M., Premkumar N., Asdaq S.M.B., Sun C.M. (2008). Synthesis and biological activities of some new fluorinated coumarins and 1-aza coumarins. Europ. J. Med. Chem..

[38] LShukla L.W.K., Moodie T., Kindahl C.J. (2018). Hedberg, Synthesis and Spectroscopic Properties of Fluorinated Coumarin Lysine Derivatives. Org. Chem..

[39] Hornick A., Lieb A., Vo N.P., Rollinger J.M., Stuppner H., Prast H. (2011). The coumarin scopoletin potentiates acetylcholine release from synaptosomes, amplifies hippocampal long-term potentiation and ameliorates anticholinergic- and age-impaired memory. Neuroscience.

[40] Solarz A., Majcher-Maślanka I., Chocyk A. (2021). Effects of early-life stress and sex on blood-brain barrier permeability and integrity in juvenile and adult rats. Dev. Neurobiol..

[41] Ronad P., Dharbamalla S., Hunshal R., Maddi V. (2008). Synthesis of novel substituted 7-(benzylideneamino)-4-methyl-2H-chromen-2-one derivatives as anti-inflammatory and analgesic agents. Arch. Pharm..

[42] Żołek T., Maciejewska D. (2017). Theoretical evaluation of ADMET properties for coumarin derivatives as compounds with therapeutic potential. Eur. J. Pharm. Sci..

[43] Wang H., Su M., Shi X., Li X., Zhang X., Yang A., Shen R. (2023). Design, Synthesis, Calculation and Biological Activity Studies Based on Privileged Coumarin Derivatives as Multifunctional Anti-AD Lead Compound. Chem. Biodivers..

[44] Orioli R., Belluti F., Gobbi S., Rampa A., Bisi A. (2024). Naturally Inspired Coumarin Derivatives in Alzheimer’s Disease Drug Discovery: Latest Advances and Current Challenges. Molecules.

[45] Hamuľaková S., Gucký A., Mezencev R., Kožurková M., Bednáriková Z., Marek J., Soukup O., Janoušek J., Gažová Z. (2025). Inhibition of amyloid fibrillization of amyloid β peptide by 4,7-disubstituted coumarin derivatives. Bioorg. Med. Chem..

[46] Sarhan M.O., Abd El-Karim S.S., Anwar M.M., Gouda R.H., Zaghary W.A., Khedr M.A. (2021). Discovery of New Coumarin-Based Lead with Potential Anticancer, CDK4 Inhibition and Selective Radiotheranostic Effect: Synthesis, 2D & 3D QSAR, Molecular Dynamics, In Vitro Cytotoxicity, Radioiodination, and Biodistribution Studies. Molecules.

[47] Sharma A., Bharate S.B. (2023). Synthesis and Biological Evaluation of Coumarin Triazoles as Dual Inhibitors of Cholinesterases and β-Secretase. ACS Omega.

[48] Huang C.-C., Chang K.-H., Chiu Y.-J., Chen Y.-R., Lung T.-H., Hsieh-Li H.M., Su M.-T., Sun Y.-C., Chen C.-M., Lin W. (2021). Multi-Target Effects of Novel Synthetic Coumarin Derivatives Protecting Aβ-GFP SH-SY5Y Cells against Aβ Toxicity. Cells.

[49] Młotkowska P., Marciniak E., Misztal A., Misztal T. (2023). Effect of Neurosteroids on Basal and Stress-Induced Oxytocin Secretion in Luteal-Phase and Pregnant Sheep. Animals.

[50] Welento J., Szteyn S., Milart Z. (1969). Observations on the stereotaxic configuration of the hypothalamus nuclei in the sheep. Anat. Anz..

[51] Haziak K., Herman A.P., Wojtulewicz K., Pawlina B., Paczesna K., Bochenek J., Tomaszewska-Zaremba D. (2018). Effect of CD14/TLR4 antagonist on GnRH/LH secretion in ewe during central inflammation induced by intra cerebro ventricular administration of LPS. J. Anim. Sci. Biotechnol..

[52] Curley P., Rajoli R.K.R., Moss D.M., Liptrott N.J., Letendre S., Owen A., Siccardi M. (2017). Efavirenz Is Predicted To Accumulate in Brain Tissue: An In Silico, In Vitro, and In Vivo Investigation. Antimicrob. Agents Chemother..

[53] Visentin M., Chang M.-H., Romero M.F., Zhao R., Goldman I.D. (2012). Substrate- and PH-Specific Antifolate Transport Mediated by Organic Anion-Transporting Polypeptide 2B1 (OATP2B1-SLCO2B1). Mol. Pharmacol..

[54] Glaeser H., Bailey D.G., Dresser G.K., Gregor J.C., Schwarz U.I., McGrath J.S., Jolicoeur E., Lee W., Leake B.F., Tirona R.G. (2007). Intestinal Drug Transporter Expression and the Impact of Grapefruit Juice in Humans. Clin. Pharmacol. Ther..

[55] Milovanovic V., Minic R., Vakic J., Ivanovic S., Cupic V., Borozan S., Nesic A., Zivkovic I. (2021). MTT based L-aminoacid oxidase activity test for determination of antivenom potency against Vipera ammodytes envenomation. Toxicon.

[56] Tataringa G., Zbancioc A.M., Rao A.V. (2020). Phytochemicals in Human Health.

[57] Bandelj P., Jamnikar-Ciglenecki U., Ocepek M., Blagus R., Vengust M. (2018). Risk factors associated with fecal shedding of Listeria monocytogenes by dairy cows and calves. J. Vet. Intern. Med..

[58] Sigurdsson S., Gudbjarnason S. (2007). Inhibition of acetylcholinesterase by extracts and constituents from Angelica archangelica and Geranium sylvaticum. Z. Naturforsch C J. Biosci..

[59] Wenjie L., Limeng W., Wenwu L., Liting T., Chen Ch H., Wu Z., Wang N., Liu X., Qiu J., Feng X. (2022). Design, synthesis and biological evaluation of novel coumarin derivatives as multifunctional ligands for the treatment of Alzheimer’s disease. Eur. J. Med. Chem..

[60] van der Waterbeemd H., Camenisch G., Folkers G., Chretien J.R., Raevsky O.A. (1998). Estimation of blood-brain barrier crossing of drugs using molecular size and shape, and H-bonding descriptors. J. Drug Target..

[61] Solarz A., Majcher-Maślanka I., Kryst J., Chocyk A. (2023). Early-life stress affects peripheral, blood-brain barrier, and brain responses to immune challenge in juvenile and adult rats. Brain Behav. Immun..

[62] Pluimer B.R., Colt M., Zhao Z. (2020). G Protein-Coupled Receptors in the Mammalian Blood-Brain Barrier. Front. Cell. Neurosci..

[63] Parvez M.M., Sadighi A., Ahn Y., Keller S.F., Enoru J.O. (2023). Uptake Transporters at the Blood–Brain Barrier and Their Role in Brain Drug Disposition. Pharmaceutics.

[64] Okazaki N., Lankadeva Y.R., Peiris R.M., Birchall I.E., May C.N. (2021). Rapid and persistent decrease in brain tissue oxygenation in ovine gram-negative sepsis. Am. J. Physiol. Regul. Integr. Comp. Physiol..

[65] Disdier C., Awa F., Chen X., Dhillon S.K., Galinsky R., Davidson J.O., Lear C.A., Bennet L., Gunn A.J., Stonestreet B.S. (2020). Lipopolysaccharide-induced changes in the neurovascular unit in the preterm fetal sheep brain. J. Neuroinflamm..

[66] Garnier Y., Berger R., Alm S., von Duering M.U., Coumans A.B., Michetti F., Bruschettini M., Lituania M., Hasaart T.H., Gazzolo D. (2006). Systemic endotoxin administration results in increased S100B protein blood levels and periventricular brain white matter injury in the preterm fetal sheep. Eur. J. Obstet. Gynecol. Reprod. Biol..

[67] Finnie J.W. (2003). Pathogenesis of brain damage produced in sheep by *Clostridium perfringens* type D epsilon toxin: A review. Aust. Vet. J..

[68] Pérez-Fernández R., Cazanga V., Jeldres J.A., Silva P.P., Riquelme J., Quiroz F., Palma C., Carretta M.D., Burgos R.A. (2017). Plasma and tissue disposition of florfenicol in *Escherichia coli* lipopolysaccharide-induced endotoxaemic sheep. Xenobiotica.

[69] Traczyk W., Przekop F. (1963). A method for testing the function of the hypothalamus and pituitary in the sheep in chronic experiments. Acta Physiol. Pol..

[70] Adan H., Guy S., Arulanandam R., Geletu M., Daniel J., Raptis L. (2022). Activated Src requires Cadherin-11, Rac, and gp130 for Stat3 activation and survival of mouse Balb/c3T3 fibroblasts. Cancer Gene Ther..

[71] Orlandini M., Oliviero S. (2001). In fibroblasts Vegf-D expression is induced by cell-cell contact mediated by cadherin-11. J. Biol. Chem..

[72] Windle J.J., Weiner R.I., Mellon P.L. (1990). Cell lines of the pituitary gonadotrope lineage derived by targeted oncogenesis in transgenic mice. Mol. Endocrinol..

[73] van Kuppeveld F.J., Johansson K.E., Galama J.M., Kissing J., Bölske G., van der Logt J.T., Melchers W.J. (1994). Detection of mycoplasma contamination in cell cultures by a mycoplasma group-specific PCR. Appl. Environ. Microbiol..

[74] Shokrzadeh M., Modanloo M. (2017). An overview of the most common methods for assessing cell viability. J. Res. Med. Dent. Sci..

[75] Alarid E.T., Windle J.J., Whyte D.B., Mellon P.L. (1996). Immortalization of pituitary cells at discrete stages of development by directed oncogenesis in transgenic mice. Development.

[76] Strzetelski J., Brzóska F., Kowalski Z., Osięgłowski S. (2014). IZ PIB–INRA Feeding Recommendations for Ruminants and Feed Tables.

[77] Albrecht P., Hryniewicz W., Kuch A., Skoczyńska A., Zajkowska J. (2024). Rekomendacje Postępowania w Zakażeniach Ośrodkowego Układu Nerwowego—Leczenie, Profilaktyka, Nadzór Nad Zakażeniami.

[78] Koper-Lenkiewicz O., Kamińska J., Lewoniewska S., Wilińska E. (2018). Selektywność Transportu Przez Barierę Krew-Mózg Polski Przegląd Neurologiczny.

[79] Brzezińska K., Ziaja M. (2012). The structure and role of blood-brain barier. Postępy Biol. Komórki.

[80] Caveney N.A., Li F.K.K., Strynadka N.C.J. (2018). Enzyme structures of the bacterial peptidoglycan and wall teichoic acid biogenesis pathways. Curr. Opin. Structur. Biol..

[81] Kessell A.E., Finnieb J.W., Windsorc P.A. (2011). Neurological diseases of ruminant livestock in Australia. III: Bacterial and protozoal infections. Aust. Vet. J..

[82] MHerian Wojtas A., Maćkowiak M., Wawrzczak-Bargiela A., Solarz A., Bysiek A., Madej K., Gołembiowska K. (2022). Neurotoxicological profile of the hallucinogenic compound 25I-NBOMe. Sci. Rep..

[83] Shohami E., Novikov M., Mechoulam R. (1993). A nonpsychotropic cannabinoid, HU-211, has cerebroprotective effects after closed head injury in the rat. J. Neurotrauma..

[84] World Health Organization (WHO). https://www.who.int/news-room/fact-sheets/detail/adolescents-health-risks-and-solutions.

