# Supplementary material for: Blood–Brain Barrier Penetration of Novel 4-Trifluoromethyl-Coumarin Hybrids with Antibacterial Properties as Potential Brain Therapeutics in the Context of Spatially Diverse Healthcare Systems
Source: Int J Mol Sci. 2025 Oct 3;26(19):9655. doi: 10.3390/ijms26199655 (PMC12525028; doi:10.3390/ijms26199655)
Supplement: Supplementary file 1 [file ijms-26-09655-s001.zip › ijms-3825165-supplementary.pdf]

Supplementary Materials

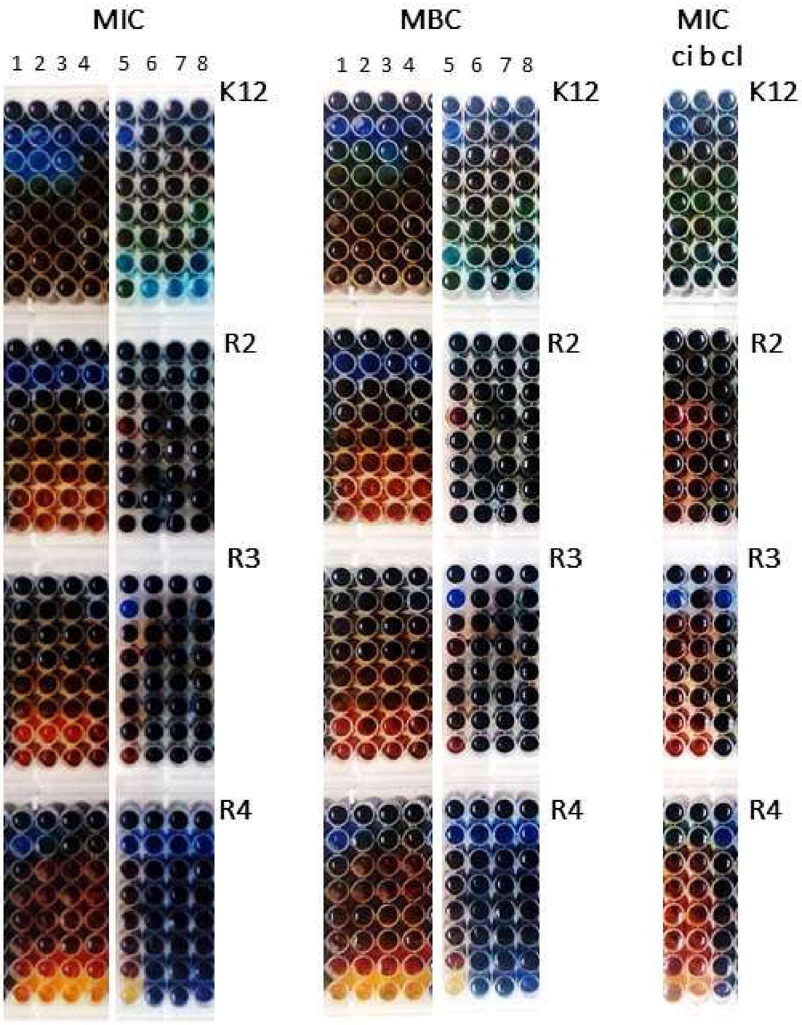

Figure S1. Examples of MIC and MBC on microplates with different concentration of studied compounds (μg/mL−1). Resazurin was added as an indicator of microbial growth with K12, R2, R3, R4 strains with tested compounds. Additionally, examples of MIC with different strains K12, R2, R3, R4 of studied antibiotics with ciprofloxacin (ci), bleomycin (b), and cloxacillin (cl) in (μg/mL−1).

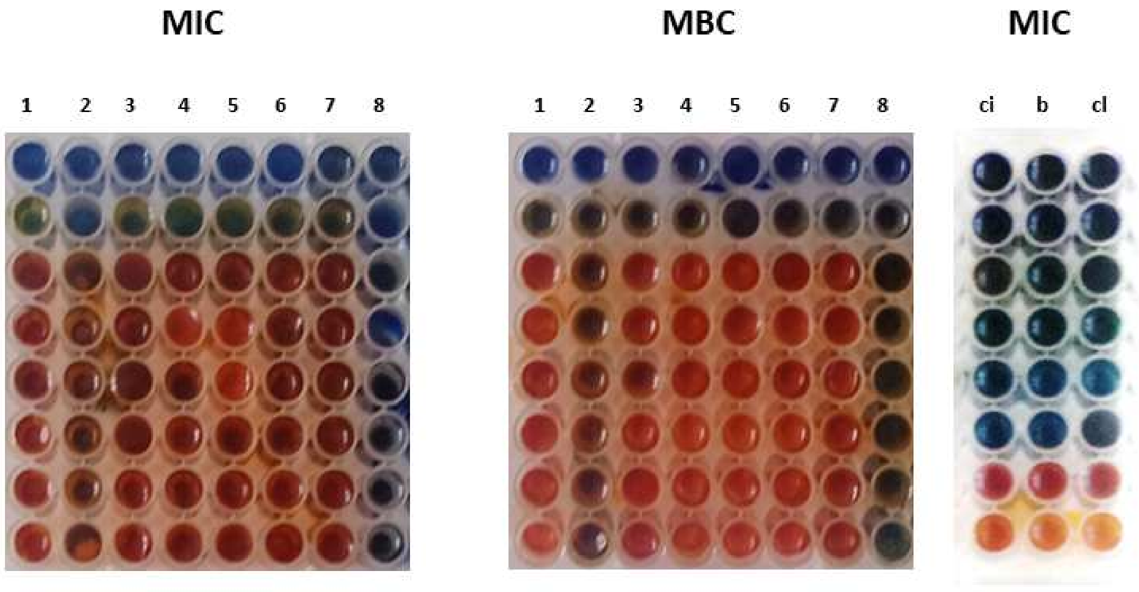

Figure S2. Examples of MIC and MBC on microplates with of studied compounds (μg/mL^{-1}). Resazurin was added as an indicator of microbial growth with *S.areus* strain Additionally, examples of MIC and MBC with different strains *S.areus* studied antibiotics with ciprofloxacin (ci), bleomycin (b), and cloxacillin (cl) in (μg/mL^{-1}).

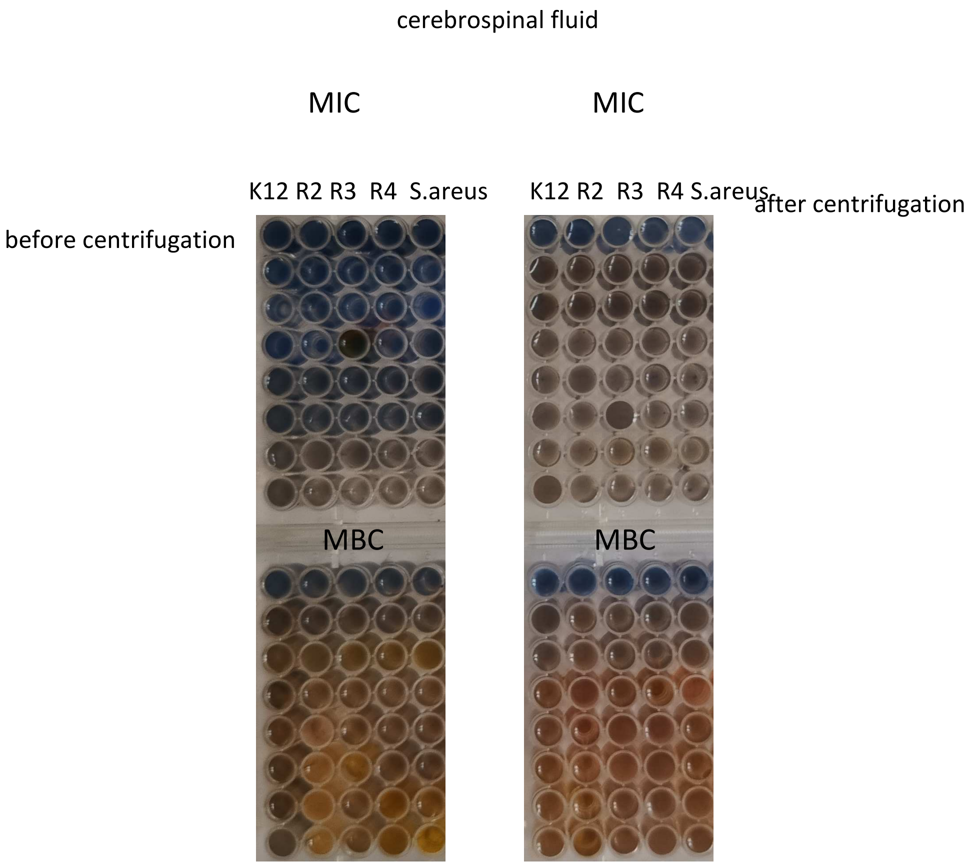

Figure S3. Examples of MIC and MBC on microplates with of studied cerebrospinal fluid (µg/mL^{-1}). Resazurin was added as an indicator of microbial growth with *E.coli* all strains and *S.areus* strain.

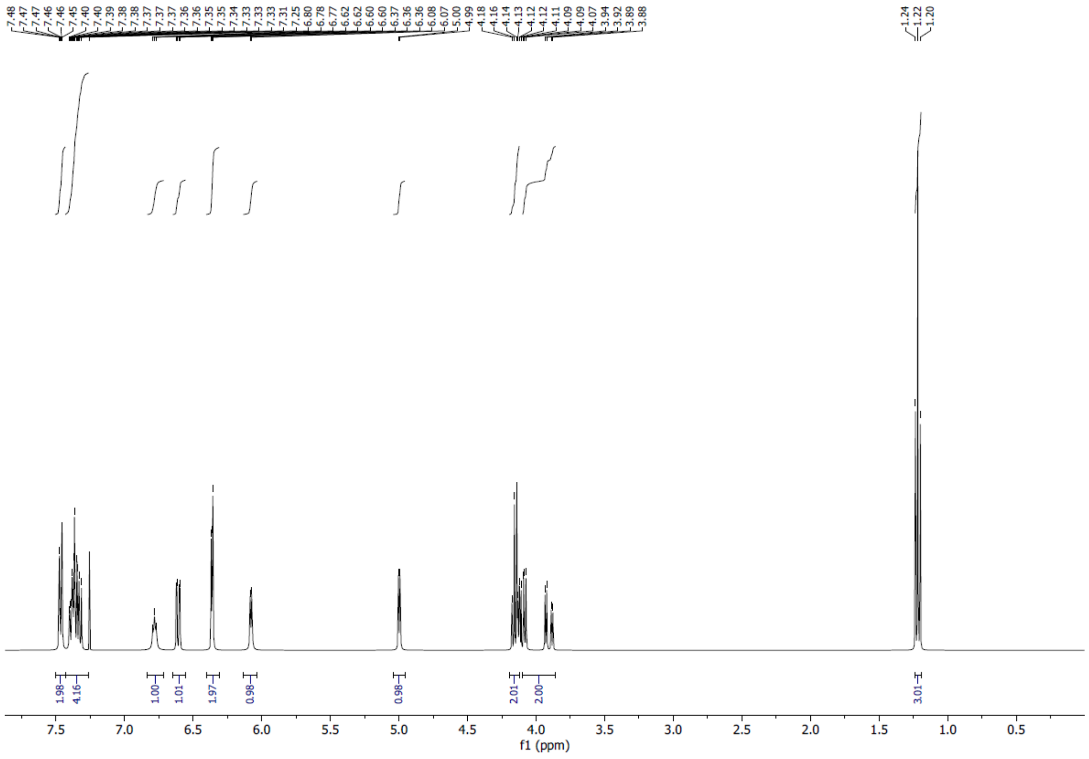

Figure S4. ^{1}HNMR (400 MHz, $CDCl_3$) spectra of 2 compound

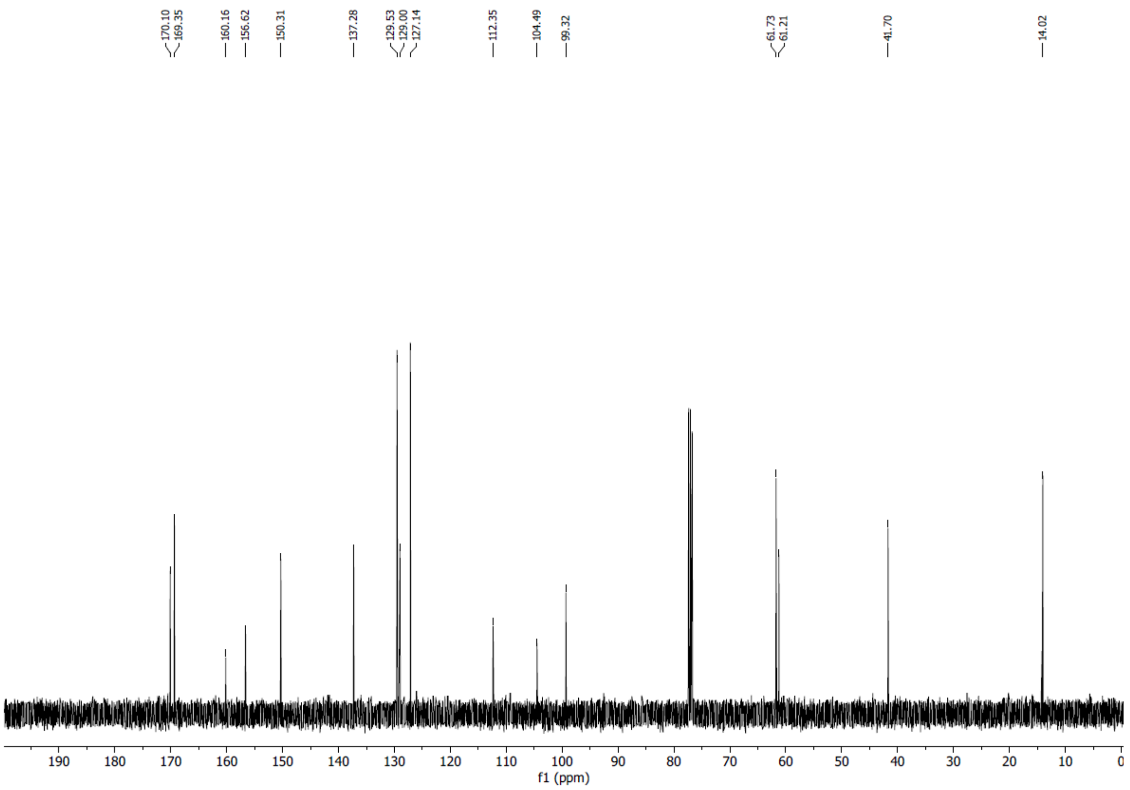

Figure S5. ^{13}CNMR (100 MHz, $CDCl_3$) spectra of 2 compound

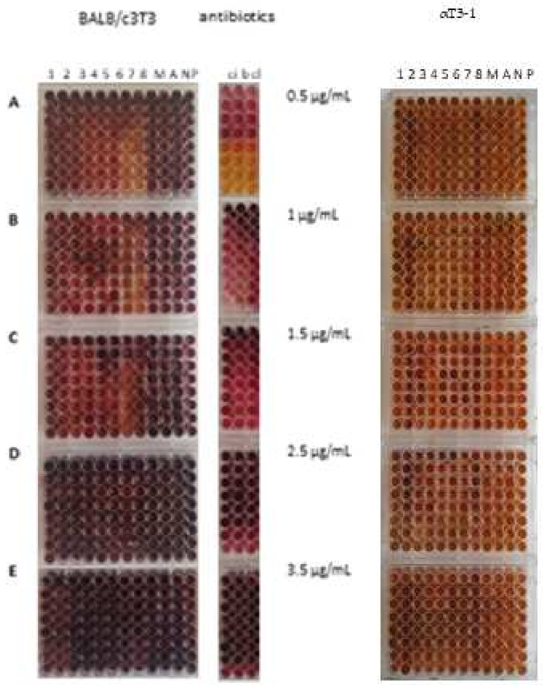

Figure S6. Configuration of the 96-well flat bottom plates in which the cytotoxicity assay was carried out on mouse embryonic fibroblast cell line BALB/C3T3 and BALB/c3T3 mouse fibroblast cells and gonadotroph cell lines such αT3-1. M= complete medium without cells. A= complete medium without extract solutions. N=negative control P=positive control. Lanes from 1-8 tested compounds at concentration 1 µg/mL each. Lanes from 1-3 tested compounds at concentration 1 µg/ml each. 1-ciprofloxacin (cipro), 2-bleomycin (bleo), 3- cloxacillin (clox).
